# The mutation spectrum and ethnic distribution of non-hepatorenal tyrosinemia (types II, III)

**DOI:** 10.1186/s13023-022-02579-0

**Published:** 2022-12-05

**Authors:** Zahra Beyzaei, Sara Nabavizadeh, Sara Karimzadeh, Bita Geramizadeh

**Affiliations:** 1 Transplant Research Center, Shiraz University of Medical Sciences, Shiraz, Iran; 2 Shiraz Medical School Library, Shiraz University of Medical Sciences, Shiraz, Iran; 3 Department of Pathology, Medical School of Shiraz University, Shiraz Transplant Research Center (STRC), Shiraz University of Medical Sciences, Khalili St., Research Tower, Seventh Floor, Shiraz, Iran

**Keywords:** Tyrosinemia, Genotype, Mutations, Tyrosine aminotransferase, 4-Hydroxyphenylpyruvate dioxygenase

## Abstract

**Background:**

Different types of non-hepatorenal tyrosinemia are among the rare forms of tyrosinemia. Tyrosinemia type II and III are autosomal recessive disorders caused by pathogenic variants in the tyrosine aminotransferase (*TAT*), and 4-hydroxyphenyl-pyruvate dioxygenas (*HPPD*) genes, respectively. There are still unclarified aspects in their clinical presentations, mutational spectrum, and genotype–phenotype correlation.

**Main body:**

In this study, we evaluated the spectrum of TAT and HHPD gene mutations in patients with tyrosinemia type II and III. Moreover, biochemical and clinical findings are evaluated to establish a genotype–phenotype relationship in the above-mentioned patients. Thirty-three TAT variants have been reported in 42 families, consisting of 21 missense variants, 5 frameshift variants, 4 nonsense variants, 2 variants that primarily cause splicing site, and 1 skipping complete exon (large deletion). The most common variant is p.Arg57Ter, causing a splicing defect, and resulting in premature termination of translation, which was found in 10 patients from 3 families. In HPPD gene, eleven variants in 16 patients have been reported including 7 missense variants, 2 nonsense variants, 1 splice defect, and 1 frameshift variant so far. All variants are unique, except for p.Tyr160Cys, which is a missense variant found in two different patients. Regarding genotype–phenotype correlations, in 90% of tyrosinemia type II patients, positive clinical and biochemical correlations with a detected variant are observed. In HPPD gene, due to the small number of patients, it is not possible to make a definite conclusion.

**Conclusion:**

This is the first large review of variants in TAT and HPPD, highlighting the wide spectrum of disease-causing mutations. Such information is beneficial for the establishment of a privileged mutation screening process in a specific region or ethnic group.

**Supplementary Information:**

The online version contains supplementary material available at 10.1186/s13023-022-02579-0.

## Introduction

Catabolism of amino acids supplies necessary nitrogen for the synthesis of crucial compounds like neurotransmitters, hormones, and energy for the cell. The liver and kidneys are the main tissues for this enzymatic pathway that its involvement depends on the nature of the amino acid. Tyrosine is a non-essential amino acid, derived directly from diet or tissue proteins or the hydroxylation of phenylalanine, which is an essential amino acid. It is a precursor in the synthesis of catecholamines, thyroid hormones, and melanin [1]. Defect in the tyrosine metabolism pathway causes increased tyrosine level and several types of related disorders.

The two least common deficiencies of tyrosine metabolism pathway are non-hepatorenal tyrosinemia, which are called tyrosinemia type II and III. Tyrosinemia type II (OMIM 276600) or oculocutaneous tyrosinemia occurs secondary to a deficiency of the cytoplasmic enzyme, tyrosine aminotransferase (TAT: EC 2.6.1.5) [2]. TAT gene contains 12 exons, which code an active protein containing 454 amino acids. The gene is located at chromosome 16q22 which is autosomal recessive. Main manifestations of this enzyme deficiency are corneal thickening, as well as palmar and plantar hyperkeratosis. Liver and kidney functions are generally normal [3].

Tyrosinemia type III (OMIM 276710) is the rarest type of deficiency in the tyrosine metabolism pathway due to a lack of enzyme 4-hydroxyphenylpyruvate dioxygenase (HPPD). The human 4-HPPD gene is located at 12q24-qter and contains 14 exons, which encode a protein containing 392 amino acids. The 4-HPPD enzyme is expressed mainly in the liver and kidney. This disorder is autosomal recessive [4]. The symptoms of tyrosinemia type III are varied, and not well characterized. Some asymptomatic patients never develop neurological signs while some patients suffer from severe neurological symptoms in childhood.

Regarding recent developments in the management of these patients, we think that it is crucial for clinicians and healthcare providers to be informed about metabolic diseases, which would be potentially beneficial for current and future mutation-targeted therapeutic options. There are some reports about Tyrosinemia type II and III, however, no clear analysis and comparison between mutations (genotype), and their phenotype has been reported so far. The objective of this review was to identify and summarize the reported mutations in the *TAT* and *HPPD* genes and their corresponding manifestations.

## Variants

### Tyrosine aminotransferase (TAT) gene

**Fig. 1:**
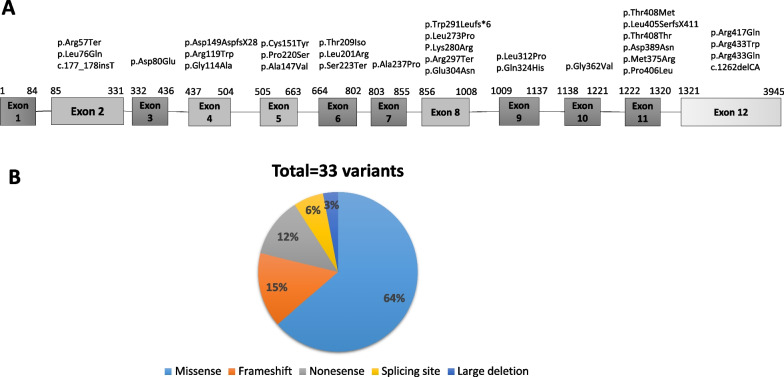
Schematic illustration (not to scale) of the TAT gene and location of sequence variants associated with Tyrosinemia type II deficiency. **A** Exon (boxes)–intron (lines) structure of the human TAT gene. Coloring boxes are consistent with panel. **B** Pie chart summarizing types of TAT variants reported to date

To date, 33 mutations in *TAT* from 42 families (74 patients) have been described to cause tyrosinemia type II (Table 1, Additional file 1: Fig. 1A) [5–17]. Missense variants illustrate the predominant variant type (n = 21) in the most recessively inherited autosomal diseases. Furthermore, 5 frameshift variants, 4 nonsense variants, 2 variants that primarily cause splicing site, and 1 skipping complete exon (large deletion) are reported (Fig. 1B). No deep intronic variants and a variant in exon 1 have been reported. The majority of variants are specific. The most frequently observed variant is p.Arg57Ter, which is a nonsense variant causing premature termination of translation, found in 10 patients from 3 families (Table 1). In all patients, this strongly inactivating variant is correlated with high-untreated plasma tyrosine levels. The variant was observed in different ethnicities including Italian, Scottish, and Native American patients. The second most frequently reported variant is p.Thr408Thr, which was found in 7 patients, of 2 unrelated Arab families (Table 1). It is a silent exonic transversion variant, which caused complete missplicing by exon 11 skipping. This skipping leads to the in-frame deletion of 99 nucleotides (33 amino acids). Therefore, the mRNA stability would be affected by this deletion. It is confirmed that this deleterious variant, is responsible for the severe manifestation in these two families. Other frequently seen missense variants are p.Cys151Tyr, p.Leu273Pro, and p.Pro406Leu, which were found in 4 patients with 2 different families, respectively (Table 1). In silico analysis of p.Cys151Tyr variant demonstrated a strong damaging effect corresponding to a deleterious impact on TAT proteins. In addition, p.Leu273Pro variant was reported to affect the stability of TAT protein either by less interaction of protein surface or the reduction of stability of tertiary structure of the protein. The rest of the variants identified in tyrosinemia type II are very rare and reported in one family (Table 1). Variants are found in all exons except exon 1, however, the distribution of missense variants across the TAT gene is not uniform (Fig. 1A). Interestingly, the check-in location of variants revealed that the first variant arises at residue 57 (p.Arg57Ter, Table 1, Fig. 1A). It is demonstrated that probably the amino acids of the N-terminal to this residue are poorly conserved. The most common accumulation of variants in this gene occurs in exon 11, which might be particularly due to the susceptibility of this region to mutation. See additional file 1 for the more information in reported patients with tyrosinemia type II and III.

**Table 1:** Summary of reported variants in *TAT* gene of patients with tyrosinemia type II

Variant	Variant type	Exon/Intron	Number of patients	Country/ethnicity	Study
p.Cys151Tyr (c.452G > A)	Missense	Exon 5	4	Tunisia	Bouyacoub et al. [5], Charfeddine et al. [6]
p.Trp291LeufsX6(c.869dupG)	Frameshift	Exon 8	1	Tunisia	Bouyacoub et al. [5],
p.Leu273Pro (c.914 T > C)	Missense	Exon 8	4	Tunisia	Charfeddine et al. [6]
p.Arg417Gln (c.1250G > A)	Missense	Exon 12	2	Croatia	Culic et al. [7], Peña-Quintana et al. [14]
p.Leu312Pro (c.935 T > C)	Missense	Exon 9	2	Turkey	Gokay et al. [8]
p.Thr408Met (c.1223C > T)	Missense	Exon 11	2	Turkey	Gokay et al. [8]
p.Leu405SerfsX411 (c.1213delCinsAG)	Frameshift	Exon 11	1	Spain	Legarda et al. [10]
p.Arg417Ter (c.1249C > T)	Nonsense	Exon 12	3	Palestinian Arab, France	Maydan et al. [11], Natt et al. [12]
p.Thr408Thr (c.1224G > T)	Silent (missplicing by exon 11 skipping)	Exon 11	7	Palestinian Arab	Maydan et al. [11]
p.Asp149AspfsX28 (c.446_ 447insA)	Frameshift	Exon 4	2	Denmark	Pasternack et al. [13]
p.Pro220Ser (c.658C > T)	Missense	Exon 5	2	Denmark	Pasternack et al. [13]
p.Arg57Ter	Nonsense	Exon 2	10	Italy, Scottish, USA	Peña-Quintana et al. [14], Huhn et al. [9], Natt et al. [12]
p.Arg119Trp	Missense	Exon 4	2	Italy	Peña-Quintana et al. [14], Huhn et al. [9]
p.Arg433Trp (c.1297C > T)	Missense	Exon 12	4	USA, Germany	Peña-Quintana et al. [14], Meissner et al. [15], Huhn et al. [9]
P.Lys280Arg	Missense	Exon 8	1	USA	Peña-Quintana et al. [14]
p.Leu76Gln	Missense	Exon 2	1	Canada French	Peña-Quintana et al. [14]
p.Ala147Val	Missense	Exon 5	2	French, Switzerland	Peña-Quintana et al. [14]
p.Thr209Iso	Missense	Exon 6	1	French	Peña-Quintana et al. [14]
p.Arg297Ter	Nonsense	Exon 8	2	Lebanon	Peña-Quintana et al. [14]
P.Ala237Pro	Missense	Exon 7	1	North Ireland	Peña-Quintana et al. [14]
p.Asp389Asn	Missense	Exon 11	2	North Ireland, England	Peña-Quintana et al. [14]
p.Met375Arg	Missense	Exon 11	1	Switzerland	Peña-Quintana et al. [14]
p.Pro406Leu	Missense	Exon 11	4	Spain (Gran Canaria), Spain	Peña-Quintana et al. [14]
p.Gly114Ala	Missense	Exon 4	2	Spain (Gran Canaria), Spain	Peña-Quintana et al. [14]
p.Gln324His	Missense	Exon 9	1	Spain	Peña-Quintana et al. [14]
p.Leu201Arg	Missense	Exon 6	1	French	Huhn et al. [9]
p.Arg433Gln	Missense	Exon 12	3	Scottish, USA	Huhn et al. [9]
c.1262delCA	Frameshift	Exon 12	1	Japan	Minami-Hori et al. [16]
p.Ser223Ter	Nonsense	Exon 6	1	Japan	Natt et al. [12]
p.Asp80Glu	Splice site	Exon 3	1	Japan	Natt et al. [12]
p.Gly362Val	Missense	Exon 10	1	France	Natt et al. [12]
p.Glu304Asn	Splice site	Exon 8	1	France	Natt et al. [12]
c.177_178insT;V60CfsX33	Frameshift	Exon 2	1	Brazil	Soares et al. [17]

Regarding the geographical distribution of variants, the majority of reported variants are patients from Europe, North America, Japan, Tunisia, and Palestinian Arabs. For the most prevalent variant in TAT gene, p.Arg57Ter, the founder effect is apparent in northern Italy, which should be analyzed in other populations of Mediterranean ancestry. Little information about the epidemiology and molecular defects in tyrosinemia type II patients from East Asia is available at this time. To the best of our knowledge, no mutation in the TAT gene has been reported from Central America, Africa, the Middle East, and the Oceania continent.

### 4-Hydroxyphenylpyruvate dioxygenase (HPPD) gene

Eleven disease-causing variants in 16 patients are recently reported in *HPPD* gene [4, 18–23]. All 11 HPPD alleles are divided into 7 missense variants, 2 nonsense variants, 1 splice defect, and 1 frameshift variant (Table 2). Nine of them are in the exonic region and 2 out of 11 variants are intronic ones. All variants are unique, except p.Tyr160Cys, which is a missense variant found in 2 different patients from 2 families (Table 2). The analysis of the crystal structure of *Pseudomonas* enzyme demonstrated that the Tyr160 residue corresponds to a position in an alpha helix, which is involved in inter-subunit contacts. Therefore, the position of variant might be important for the stability of the enzyme, but this needs to be further elucidated. Three variants were reported in exon 11 and 2 variants in the intronic region of 10, and 11. Therefore, the largest accumulation of variants happens in this region, which is suggestive of its susceptibility to mutation.

**Table 2:** Summary of reported variants in HPPD gene of patients with tyrosinemia type III

Variant	Variant type	Exon/Intron	Number of patients	Country/Ethnicity	Study
p.Ala33Thr (c.97G > A)	Missense	Exon 4	2	Portugal	Barroso et al. [4]
IVS11 + 1G > A	Splice site	Intron 11	1	Turkey	Heylen et al. [18]
p.Tyr200Ter (c.11266C > G)	Nonsense	Exon 10	1	Sweden	Rüetschi et al. [19]
p.Ile335Met (c.18470C > G)	Missense	Exon 13	1	Sweden	Rüetschi et al. [19]
p.Tyr160Cys (c.479A > G)	Missense	Exon 8	3	Sweden, Poland	Rüetschi et al. [19], Szymanska et al. [20]
p.Tyr258Ter (c.11558T > G)	Nonsense	Exon 11	2	Sweden	Rüetschi et al. [19]
p.Ile267Phe (c.11583A > T)	Missense	Exon 11	2	Sweden	Rüetschi et al. [19]
p.Ala268Val (c.803C > T)	Missense	Exon 11	1	Japan	Tomoeda et al. [21]
c.759 + 1 G > A	Missense	Intron 10	1	Iran	Vakili et al. [22]
p.Gly154Ser (c.460G > A)	Missense	Exon 8	1	China	Zhao et al. [23]
p.Gly83Ter (c.248delG)	Frameshift	Exon 5	1	China	Zhao et al. [23]

Regarding the geographical distribution of variants, the majority of reported variants are patients from Europe, and Asia (Japan, China, and Iran). No information about the epidemiology and molecular defects in tyrosinemia type III patients from North and Central America, Africa, Australia, and the Oceania continent are available.

### Genotype–phenotype correlation

The correlation between genotype and phenotype is described as the possibility of an association between a specific mutation and/or class of mutation with a special clinical abnormality. Therefore, finding genotype–phenotype correlations in inherited metabolic disorders as rare diseases are difficult and complicated by several factors, such as the absence of large cohorts of patients for analysis, the large phenotypic heterogeneity associated with the same mutation, and a high proportion of private mutations [24]. The great number of variants in TAT and HPPD genes are private variants, so, genotype–phenotype correlations or correlations between type or location of the mutation and clinical manifestation have not been established so far. Therefore, for this purpose, we present a comprehensive list of patients identified from the reported literature, along with clinical and biochemical data (Additional file 1: Table S1). This helped us not only to precisely estimate the number of published patients with tyrosinemia type II and III diseases but also helped to stratify patients for genotype–phenotype correlations.

Data on clinical and biochemical results of tyrosinemia type II were available for 70 patients. We have identified among them, only 7 patients had elevated levels of tyrosine along with detected pathogenic variants, however, clinical manifestations were asymptomatic (Additional file 1: Table S1). Approximately 90% of patients had correlated clinical and biochemical manifestations with the type of detected variant.

Nevertheless, 5 out of 16 patients from 4 different families and populations with tyrosinemia type III were asymptomatic even though they had elevated tyrosine levels and high urinary excretion of 4-HPL and 4-HPP. However, due to the small number of patients, it is not possible to conclude clearly. It needs further investigation.

## Conclusion

In conclusion, this report allows a detailed identification of the variants causing non-hepatorenal tyrosinemia (tyrosinemia type II, III) for future carrier and prenatal screening. It would be useful for clinicians to focus on specific variants of definite regions, which facilitates the targeted detection of disease. Although not all cases of non-hepatorenal tyrosinemia are described in the literature, establishing a genotype–phenotype correlation is difficult. Although the pathophysiology of non-hepatorenal tyrosinemias is not completely explained, it seems that multicenter collection of data and further studies on these patients are necessary to understand the consequences of these deficiencies, the mechanisms of injuries, and the long-term outcome of these patients.

## Supplementary Information

**Additional file 1. Table 1.** Summary of reported patients with tyrosinemia type. **Table 2.** Summary of reported patients with tyrosinemia type III.

## Data Availability

The data generated for this study are available upon reasonable request from the corresponding author.

## Abbreviations

HPPD: 4-Hydroxyphenylpyruvate dioxygenase
TAT: Tyrosine aminotransferase
4-HPL: 4-Hydroxyphenyllactic acid
4-HPP: 4-Hydroxyphenylpyruvic acid
