# Supplementary material for: The mutation spectrum and ethnic distribution of non-hepatorenal tyrosinemia (types II, III)
Source: Orphanet J Rare Dis. 2022 Dec 5;17:424. doi: 10.1186/s13023-022-02579-0 (PMC9724276; doi:10.1186/s13023-022-02579-0)
Supplement: Supplementary file 1 — Additional file 1. Table 1. Summary of reported patients with tyrosinemia type. Table 2. Summary of reported patients with tyrosinemia type III. [file 13023_2022_2579_MOESM1_ESM.pdf]

TABEL 1. Summary of reported patients with tyrosinemia type II

Study	Country/ Ethnicity	Gender	Age of diagnosis	Biochemical findings at onset	Clinical Manifestation
**Bouyacoub et al. 2013**	Tunisian	M	9 month	elevated tyrosine levels (1750 µmol/L), high 4-HPL in urine	An erythematous, painful focal palmoplantar hyperkeratosis, geographic tongue, a mild developmental retardation
		M	4 month	elevated tyrosine levels (1725 µmol/L), high urinary 4-HPL, lactic acids, N-acetyl-tyrosine	Eye redness, photophobia, tearing, pain and bilateral pseudo dendritic keratitis
**Charfeddine et al. 2006**	Tunisia	F	9 years	elevated tyrosine levels (1255 µmol/L),	Painful palmar and plantar keratosis, mild mental impairment, a mild language disorder
		F	8 years	elevated tyrosine levels (915 µmol/L),	Painful palmar and plantar keratosis, mild mental impairment, a mild language disorder (eye redness improved spontaneously in summer)
		F	6 years	elevated tyrosine levels (893 µmol/L),	Painful palmar and plantar keratosis, a decreased visual acuity, a history of recurrent “red eyes” without lacrimination, mild mental impairment, a mild language disorder (Skin symptoms improved spontaneously in summer)
		M	4 month	elevated tyrosine levels (760 µmol/L),	Bilateral keratitis
		F	Infancy	-	Photophobia, a painful focal palmoplantar hyperkeratosis
		F	Infancy	-	Photophobia, a painful focal palmoplantar hyperkeratosis
		M	4 month	elevated tyrosine levels (1939 µmol/L), high urinary 4-HPL, N-acetyl tyrosine	Bilateral keratitis, a mild psychomotor developmental delay with hemiparesis and seizures
**Culic et al. 2011**	Croatia	F	8 years	elevated tyrosine levels (1879 µmol/L), high urinary 4-HPL	Photophobia, agitation, focal hyperkeratosis on the palms and soles, hyperkeratosis, slightly hypotonic, abnormal EEG
**Gokay et al. 2016**	Turkey	M*	4 years	elevated tyrosine levels (1450 µmol/L), high urinary excretion of 4-HPL and 4-HPP, n-acetyl tyrosine	Photophobia, mild mental retardation, abnormal EEG
		F*	1 year	elevated tyrosine levels (1576 µmol/L)	Asymptomatic
**Legarda et al. 2011**	Spain	F	6 months	elevated tyrosine levels (1038 µmol/L), high urinary excretion of 4-HPL and 4-HPP	Dendritic keratitis and painful palmar hyperkeratosis, photophobia
**Maydan et al. 2006**	Palestinian Arab	M*	9 years	elevated tyrosine levels (1261 µmol/L),	Painful bilateral palmoplantar hyperkeratoses
		M*	14 months	elevated tyrosine levels (1366 µmol/L),	Developed skin manifestations by age
		M†	20 months	elevated tyrosine levels (1280 µmol/L),	Bilateral pseudo dendritic keratitis
		F†	3 days	elevated tyrosine levels (708 µmol/L),	Asymptomatic, newborn
		F€	21 years	elevated tyrosine levels (515 µmol/L),	Asymptomatic,

		F€	27 years	elevated tyrosine levels (814 µmol/L),	Chronic skin and eye lesions
		M	11 months	elevated tyrosine levels (1579 µmol/L),	NR
		M®	25 years	elevated tyrosine levels (1024 µmol/L),	Severe ocular and skin manifestations, moderately mentally retarded
		M®	30 years	elevated tyrosine levels (1098 µmol/L),	Skin manifestations
**Pasternack et al. 2008**	Denmark	F	28 years	elevated tyrosine levels (1636 µmol/L),	Severe nail dystrophy, developed palmar hyperkeratosis, eye pain and photophobia
		M	30 years	elevated tyrosine levels (1112 µmol/L),	Mild hyperkeratosis,
**Peña-Quintana et al. 2017**	Italy	M	1 month	elevated tyrosine levels (800 µmol/L),	Eye and skin problem
	USA	M	NR	elevated tyrosine levels (1500 µmol/L),	Eye and skin problem
	Canada French	F	NR	NR	NR
	French	M	17 years	elevated tyrosine levels (1587 µmol/L),	Eye, skin, and mild neurologic problem
	Lebanon	M	8 Months	elevated tyrosine levels (1546 µmol/L),	Eye and skin problem
	Lebanon	M	4 days	elevated tyrosine levels (2442 µmol/L),	Asymptomatic
	N. Ireland	F	NR	elevated tyrosine levels (1000 µmol/L),	Asymptomatic
	Switzerland	F	6 months	elevated tyrosine levels (2229 µmol/L),	Eye problem developmental delay
	English	F	7 days	elevated tyrosine levels (1809 µmol/L),	Mild skin problem,
	Croatian	F	12 days	elevated tyrosine levels (1139 µmol/L),	Asymptomatic
	Spain (Gran Canaria)	M	11 months	elevated tyrosine levels (836 µmol/L),	Eye, skin, and neurologic problem
	Spain (Gran Canaria)	M	11 months	elevated tyrosine levels (1092 µmol/L),	Eye, skin, and neurologic problem
	Spain (Gran Canaria)	M	6 months	elevated tyrosine levels (2750 µmol/L),	Photophobia., skin, and neurologic problem
	Spain (Gran Canaria)	M*	4 years	elevated tyrosine levels (680 µmol/L),	Photophobia., skin, and mild neurologic problem
	Spain (Gran Canaria)	M*	NR	elevated tyrosine levels (1731 µmol/L),	Skin, and mild neurologic problem, photophobia,
	Spain	F		elevated tyrosine levels (1030 µmol/L),	Keratitis
	Spain	M	13 years	elevated tyrosine levels (680 µmol/L),	Eye, skin, and neurologic problem
**Huhn et al. 1998**	Italian	M*		elevated tyrosine levels (1250 µmol/L),	Photophobia, slight mental retardation

		F*	NR	elevated tyrosine levels (1050 µmol/L),	NR
	Italian	M	29 years	elevated tyrosine levels (800 µmol/L),	Eye and skin lesions
		F	27 years	elevated tyrosine levels (850 µmol/L),	Eye and skin lesions
		M	3 months	elevated tyrosine levels (1400 µmol/L),	Hyperkeratosis
	French	F	2 months	elevated tyrosine levels (1300 µmol/L),	Severe photophobia, Bilateral keratitis
	Scottish	M*	Few months	elevated tyrosine levels (1300 µmol/L),	Photophobia, failed to thrive
		F*	18 days	elevated tyrosine levels (700 µmol/L),	Photophobia,
	USA	M	11 months	elevated tyrosine levels (1500 µmol/L),	Photophobia, bilateral dendritic keratitis, and hyperkeratotic lesions
**Meissner et al. 2008**	Germany	F*	Newborn screening	elevated tyrosine levels (1430 µmol/L), high urinary excretion of 4-HPL and 4-HPP	Asymptomatic
		M*	8 years	elevated tyrosine levels (806 µmol/L)	Hyperkeratotic plaques
**Minami-Hori et al. 2005**	Japan	M	58 years	elevated tyrosine levels (1560 µmol/L) high urinary excretion of 4-HPL and 4-HPP	Slight dementia, palmoplantar keratosis, ophthalmalgia, bilateral cataracts and severe mental retardation
**Natt et al. 1992**	Japan	F	2.6 years	elevated tyrosine levels (1118 µmol/L) high urinary excretion of 4-HPL and 4-HPP	Photophobia, tenderness on palms and soles
	France	NR	NR	NR	NR
	Italy	F	3 years	elevated tyrosine levels (1210 µmol/L)	Eye and skin lesions
**Soares et al. 2006**	Brazil	M	2 years	elevated tyrosine levels (1004 µmol/L)	Plantar hyperkeratosis

* † € ® Siblings

HPL, 4-hydroxy phenolic acids; NR, not reported

TABLE 2. Summary of reported patients with tyrosinemia type III

Study	Country/ Ethnicity	Gender	Age of diagnosis	Biochemical findings at onset	Clinical Manifestation
**Barrosoa et al. 2020**	Portugal	M*	Neonatal screening	elevated tyrosine levels (526 µmol/L), high urinary excretion of 4-HPL and 4-HPP and presence of N-acetyl-tyrosine and vanillactic acid	Language development progressed slowly, with sound articulation problems, a hyperactive behavior, ADHD
		F*	8 years	elevated tyrosine levels (1769 µmol/L),	Primary nocturnal enuresis and vesical instability, ADHA
**Heylen et al. 2012**	Turkey	M	Neonatal screening	elevated tyrosine levels (398 µmol/L), high urinary excretion of 4-HPL and 4-HPP	Persistence of jaundice
**Rüetschi et al. 2000**	Sweden	M	8 months	elevated tyrosine levels (530 µmol/L), high urinary excretion of 4-HPL and 4-HPP	Developmental delay, severe mental retardation (IQ 48), abnormal behavior, and neurological abnormalities
		M*	18 years	elevated tyrosine levels (230 µmol/L), high urinary excretion of 4-HPL and 4-HPP	Slight psychomotor retardation
		M*	7.5 years	elevated tyrosine levels (830 µmol/L), high urinary excretion of 4-HPL and 4-HPP	Slight psychomotor retardation
		F	Neonatal screening	elevated tyrosine levels (1094 µmol/L), high urinary excretion of 4-HPL and 4-HPP	Developmental delay
		M	Neonatal screening	elevated tyrosine levels (1000 µmol/L), high urinary excretion of 4-HPL and 4-HPP	Asymptomatic
**Szymanska et al. 2015**	Poland	F	7 years	elevated tyrosine levels (535 µmol/L), high urinary excretion of 4-HPL and 4-HPP	Asymptomatic
**Tomoeda et al. 2000**	Japan	M	16 years	elevated tyrosine levels, high urinary excretion of 4-HPL and 4-HPP	Microencephaly and mild mental retardation
**Vakili et al. 2020**	Iran	F	8 years	elevated tyrosine levels (633 µmol/L), high urinary excretion of 4-HPL and 4-HPP	Asymptomatic
**Zhao et al. 2020**	China	NR	Newborn screening	elevated tyrosine levels (732 µmol/L),	Asymptomatic
		NR	Newborn screening	elevated tyrosine levels (715 µmol/L),	Asymptomatic

*Siblings; ADHD, Attention Deficit Disorder with Hyperactivity; 4-HPL, 4-hydroxy phenolic acids; 4-HPP, 4-hydroxyphenylpyruvate (4-HPP);
